# Scientific Findings of the Southern and Central Africa International Center of Excellence for Malaria Research: Ten Years of Malaria Control Impact Assessments in Hypo-, Meso-, and Holoendemic Transmission Zones in Zambia and Zimbabwe

**DOI:** 10.4269/ajtmh.21-1287

**Published:** 2022-10-13

**Authors:** Matthew M. Ippolito, Mary E. Gebhardt, Ellen Ferriss, Jessica L. Schue, Tamaki Kobayashi, Mike Chaponda, Jean-Bertin Kabuya, Mbanga Muleba, Monicah Mburu, Japhet Matoba, Michael Musonda, Ben Katowa, Mukuma Lubinda, Harry Hamapumbu, Limonty Simubali, Twig Mudenda, Amy Wesolowski, Timothy M. Shields, Andre Hackman, Clive Shiff, Maureen Coetzee, Lizette L. Koekemoer, Shungu Munyati, Lovemore Gwanzura, Susan Mutambu, Jennifer C. Stevenson, Philip E. Thuma, Douglas E. Norris, Jeffrey A. Bailey, Jonathan J. Juliano, Gershom Chongwe, Modest Mulenga, Edgar Simulundu, Sungano Mharakurwa, Peter C. Agre, William J. Moss, for the Southern and Central Africa International Center of Excellence for Malaria Research

**Affiliations:** ^1^ Johns Hopkins University School of Medicine, Baltimore, Maryland;; ^2^ Johns Hopkins Bloomberg School of Public Health, Baltimore, Maryland;; ^3^ Tropical Diseases Research Centre, Ndola, Zambia;; ^4^ Macha Research Trust, Choma, Zambia;; ^5^ Wits Research Institute for Malaria, Faculty of Health Sciences, University of the Witwatersrand and National Institute for Communicable Diseases, Johannesburg, South Africa;; ^6^ Biomedical Research and Training Institute, Harare, Zimbabwe;; ^7^ University of Zimbabwe Faculty of Medicine and Health Sciences, Harare, Zimbabwe;; ^8^ Africa University, Mutare, Zimbabwe;; ^9^ Brown University, Providence, Rhode Island;; ^10^ University of North Carolina School of Medicine, Chapel Hill, North Carolina;; ^11^ University of Zambia, Lusaka, Zambia;; ^12^ Directorate of Research and Postgraduate Studies, Lusaka Apex Medical University, Lusaka, Zambia

## Abstract

For a decade, the Southern and Central Africa International Center of Excellence for Malaria Research has operated with local partners across study sites in Zambia and Zimbabwe that range from hypo- to holoendemic and vary ecologically and entomologically. The burden of malaria and the impact of control measures were assessed in longitudinal cohorts, cross-sectional surveys, passive and reactive case detection, and other observational designs that incorporated multidisciplinary scientific approaches: classical epidemiology, geospatial science, serosurveillance, parasite and mosquito genetics, and vector bionomics. Findings to date have helped elaborate the patterns and possible causes of sustained low-to-moderate transmission in southern Zambia and eastern Zimbabwe and recalcitrant high transmission and fatality in northern Zambia. Cryptic and novel mosquito vectors, asymptomatic parasite reservoirs in older children, residual parasitemia and gametocytemia after treatment, indoor residual spraying timed dyssynchronously to vector abundance, and stockouts of essential malaria commodities, all in the context of intractable rural poverty, appear to explain the persistent malaria burden despite current interventions. Ongoing studies of high-resolution transmission chains, parasite population structures, long-term malaria periodicity, and molecular entomology are further helping to lay new avenues for malaria control in southern and central Africa and similar settings.

## INTRODUCTION

Malaria varies in endemicity across southern and central Africa according to the local ecology, level of rural development, penetration of control measures, and factors relating to the parasite, mosquito, and human host. As elsewhere in sub-Saharan Africa, *Plasmodium falciparum* is the predominant parasite species, accounting for > 98% of malaria cases. Since 2002, the Johns Hopkins Malaria Research Institute has maintained a field site and laboratory in southern Zambia through a collaboration with the Macha Research Trust (MRT) located in Choma District, Southern Province, and supported by the Bloomberg Philanthropies. With the launch of the International Centers of Excellence for Malaria Research (ICEMR) program in 2010 by the National Institute of Allergy and Infectious Diseases, the Choma District research site and two others were established as the Southern and Central Africa ICEMR with the objective of evaluating ongoing and future control and elimination efforts in each transmission setting. The second site is in a holoendemic border area in Nchelenge District, Luapula Province of northern Zambia, in partnership with the Tropical Diseases Research Centre and the third site is in a mesoendemic border area of Mutasa District, Manicaland Province in eastern Zimbabwe, in partnership with the Biomedical Research and Training Institute, Africa University, and the National Institutes of Health Research.

The background epidemiology and entomology of the three study sites are described in Table 1. Choma District in Southern Province, Zambia was once a high burden area that experienced a dramatic decline in malaria in 2007–2008 followed by sustained, low-level transmission (< 1% parasite prevalence) that continues unchanged to the present. Nchelenge District in Luapula Province, Zambia records among the highest parasite prevalence and malaria case fatality countrywide despite once having achieved near-universal insecticide treated net (ITN) coverage and conducting annual targeted indoor residual spray (IRS) campaigns during Zambia’s first two National Malaria Strategic Plans. A recent influx of over 15,000 refugees from malarious areas of the Democratic Republic of the Congo (DRC) in 2018 into the district added to the malaria burden. The third site, Mutasa District, is located in the province of Manicaland, Zimbabwe on the border with Mozambique. It is a lowland area with mesoendemic malaria that persists despite comprehensive ITN and IRS implementation.

**Table 1:** Malaria Epidemiology at the Main Study Sites of the Southern and Central Africa International Center of Excellence for Malaria Research

	Zambia		Zimbabwe
Characteristic	Choma	Nchelenge	Mutasa
District population	148,000	296,000	169,800
Predominant *Plasmodium *species	*P. falciparum*	*P. falciparum*	*P. falciparum*
Endemicity	Hypoendemic	Holoendemic	Mesoendemic
Seasonality	Single	Perennial	Single
Parasite prevalence, %*			
2012	0.7	30.8	6.4
2020	1.6	44.4	4.9
Dominant vector sp.	*An. arabiensis*	*An. funestus*	*An. funestus*
Secondary vector spp.	*An. squamosus* *An. coustani* *An. rufipes*	*An. gambiae*	*An. gambiae*
Entomological inoculation rate†	< 1	81	10

* Measured by polymerase chain reaction (Choma) or rapid diagnostic test (other sites) in all ages.

† Number of infectious bites per person per year.

The first main objective of the ICEMR is to measure spatiotemporal changes in *P. falciparum* parasitemia and serological responses in humans in these three distinct epidemiological settings in different phases of malaria control to inform tailored, effective, and efficient control strategies. To accomplish this, observational studies were carried out at population- and individual-level scales using a multidisciplinary approach that drew from classical epidemiology, geospatial science, and serosurveillance to investigate correlations, risk factors, and trends in malaria epidemiology across the spectrum of endemicity and in response to different combinations and variations of malaria control interventions.

The second main objective is to characterize the mosquito vectors at each site in terms of their bionomics, feeding and resting behaviors, insecticide susceptibility, infection rates, and population genetics. Knowledge of the anopheline vectors is vital to selecting effective intervention packages and understanding why certain vector control measures might fail.

The third main objective is to apply molecular tools to investigate the underlying dynamics of malaria epidemiology at each site. High-resolution profiles of parasite genetic diversity can reveal transmission networks, parasite population structure, and changes in parasite genetics in response to interventions.

This article summarizes the key scientific findings over the past decade of the program across the three main study sites and objectives. Because of the disparate endemicities across the study region and the distinct designs and methodologies tailored to them, the article is arranged according to geographic site. Serological studies of human populations and genetic studies of parasites spanned multiple sites. Overarching lessons and syntheses of findings across the whole study region are dealt with in the conclusion section that follows the site-specific sections. Ten years of observations yielded essential insights into how malaria transmission is perpetuated in low-, moderate-, and high-burden areas and highlight the limitations of current tools as well as opportunities and threats to malaria control.

This summary coincides with the conclusion of Zambia’s first National Malaria Elimination Strategic Plan, launched in 2017. Despite intensive scaleup of efforts and support from foreign government and nongovernmental donors, the country did not achieve its goal of eliminating malaria by 2021. Scientific findings of the ICEMR provide insight into how malaria transmission was sustained in both high- and low-transmission settings and inform the next iteration of control efforts. Zimbabwe set a more modest goal of malaria elimination by 2030 with persistent areas of mesoendemic malaria concentrated in the lowlands and international border with Mozambique, areas where ICEMR studies are focused. This summary also coincides with the recent approval by the WHO of the first malaria vaccine RTS,S/AS01. With the addition of a vaccine to the existing armamentarium of malaria control—artemisinin-based combination therapy (ACT)-based case management, IRS, and ITNs—it is timely to survey the current landscape and consider the performance of existing efforts and how they might be augmented for greater gains against malaria.

## SUSTAINED LOW-LEVEL MALARIA TRANSMISSION IN SOUTHERN ZAMBIA

Choma District is an area of seasonal hypoendemic malaria transmitted mainly by *An. arabiensis* during a rainy season lasting November to April.^1^^,^^2^ *P. falciparum* is the predominant parasite species, but *P. malariae* circulates in low numbers and infection with *P. ovale* and *P. vivax* occur rarely.^3^ The 2,000 km^2^ study area is within the catchment of Macha Mission Hospital and hosts a population of approximately 56,000 who reside mainly in homesteads scattered throughout the rural countryside.

### Methods.

Preceding the ICEMR, in 2008 MRT implemented a passive health facility–based case detection system in the area that was subsequently incorporated into the ICEMR program.^4^ Health centers report weekly malaria case counts measured by the number of positive rapid diagnostic tests (RDTs), the number of total RDTs consumed, and the number of prescribed ACT treatment courses stratified by age group (< 1 year, 1 to < 5 years, and ≥ 5 years old). Inventories of RDTs are also reported to track stockouts and account for their impact on case frequency estimations.

Four prospective observational designs were used. From 2008 to 2014, randomly selected households within the 2,000 km^2^ study area were assigned to a longitudinal cohort (*n* = 872) or serial cross-sectional surveys (*n* = 2,069) visited bimonthly for studies of parasite prevalence and household- and individual-level risk factors.^3^^,^^5^^–^^9^ In 2014, the study design was modified to assess a reactive test-and-treat (RTAT) program implemented by the National Malaria Elimination Program (NMEP). Individuals residing in index households (*n* = 676) and secondary households within a 250-m radius (*n* = 864) were enrolled and followed through July 2018.^10^^–^^12^ From September 2018 to September 2020 a longitudinal cohort study (*n* = 1,116) was conducted to assess sources of focal transmission in a proscribed study area encompassing the catchment area of a single health center with an expected high number of incident malaria cases.

Participants were administered survey questionnaires and finger prick blood samples were collected as thick and thin smear preparations and as dried blood spots on filter paper (Whatman 903TM Protein Saver Card; Sigma-Aldrich, Piscataway, NJ) for subsequent detection of parasitemia by quantitative polymerase chain reaction (qPCR), serologic assays, and parasite genotyping. *P. falciparum* specific HRP-2 antigen based RDTs (SD Bioline, Gyeonggi-do, Republic of Korea) were administered to all participants. A subset of individuals (*n* = 69) was recruited in 2013–2014 to participate in a GPS logger study of human movement patterns and malaria risk. Methods for the collection of remotely sensed data and assays for molecular and serological data are discussed in detail in the cited work.

Entomological studies had been ongoing in Choma District since 2004. These were primarily small-scale studies to identify vector species and foraging behaviors using mosquito collection methods including CDC light traps (John W Hock Co., Gainesville, FL), permethrin-spray catches, animal-baited traps, human landing collections, and aspiration collections indoors. Subsequent collections have primarily used CDC light traps and were expanded to include outdoor spaces.

### Epidemiological trends.

**Figure 1.**
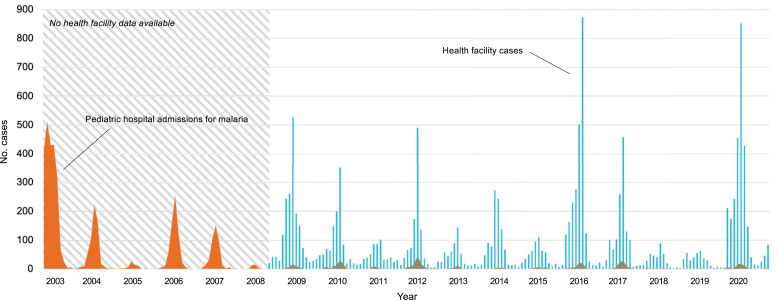
Epidemic curves of malaria in Choma District showing monthly pediatric hospital admissions for malaria and health facility malaria cases. Health facility surveillance data collection began in August 2008. The median age of children under 5 years hospitalized for severe malaria increased from 2 years in 2003 to 4 years in 2020. The proportion of health facility cases in children under 5 years decreased from 31% in 2008–2010 to 14% in 2018–2020. This figure appears in color at www.ajtmh.org.

Historically, Choma District was an area of high malaria burden. Over 3 years from 2007 to 2009, the district saw a steep decline in transmission, with parasite prevalence by RDT decreasing from 24% to 1%, coinciding with drought and scale-up of malaria control interventions.^3^ Malaria case management was updated 3 years prior, in 2004, with the introduction of artemether-lumefantrine and 1 year later the Zambian government began to supply health facilities with RDTs. In 2007, widescale ITN distributions led to net ownership in the study area of > 80%.^8^^,^^13^ Indoor residual spray campaigns were not part of the control program in Choma District, but IRS was done in targeted fashion in other parts of Southern Province and scattered parts of the district. However, since the decline in malaria transmission, parasite prevalence has remained at ∼1% to 2% in the study area. Pediatric hospitalizations for malaria similarly dropped precipitously from > 1,000 to < 50 admissions per year and remain low (Figure 1).^7^

The paucity of data in the pre-ICEMR period limited the ability to investigate the cause of the decline. It is thought to have resulted from a combination of drought in 2004–2005 that locally eradicated the *An. funestus* mosquito population in the study area and the simultaneous scale-up of malaria control interventions.^2^ Zambia also experienced a period of rapid economic growth with a steep rise in gross domestic product from US$3.6 billion in 2000 to 20.2 billion in 2010.^14^ Over this time, we observed significant increases in electrification, indoor plumbing, and quality of building materials within study households^9^ and improved road conditions. While malaria incidence remains low overall, periodic peaks in cases detected at health facilities occur. Predictive modeling of a peak in 2016 showed weak but significant correlations with rainfall and temperature patterns.^15^

Consistent with the decline in malaria cases observed at health facilities across the region, parasite prevalence in ICEMR surveyed households was low across all age groups. As transmission declined the median age of *P. falciparum*–infected individuals increased from 9 to 13 years.^6^ A similar trend was seen in the median age of children younger than 5 years hospitalized for malaria, which increased from 2.5 years in 2003–2007 to 4 years in 2017–2020 (Kruskal–Wallis *P* < 0.001), and the proportion of health facility cases in children under 5 years, which decreased from 31% in 2008–2010 to 14% in 2018–2020 (Kruskal–Wallis *P* < 0.001) (unpublished data). More than 3,000 community members were tested by qPCR to identify 61 (2%) *P. falparum* infections, all of which were low density (≤ 2,000 copies/μL) and nearly half of which (47%) were undetectable by RDT.^3^ During the low transmission dry season when parasite densities tended to be lowest, RDTs were only 17% sensitive.^3^

### Malaria risk factors.

Risk factors for *P. falciparum* infection were typical for an area with endemic malaria: younger age, sleeping without a mosquito net, low-quality housing, higher household occupancy, and open water sources near the home.^6^^,^^9^^,^^16^ Insecticide-treated net use was protective, as was residing in a higher quality house (i.e., brick or cement walls and metal roof).^9^ Children and adults had equally low prevalence (0.7%) of asexual *P. falciparum* parasitemia, but notably, gametocytes—responsible for malaria transmission—were most prevalent in school-age children 5 to 15 years old (∼2%), the group reporting the least ITN usage.^8^^,^^17^

Geospatial risk maps derived from remotely sensed data and ICEMR field data found that malaria tends to be concentrated in areas nearby streams at lower elevations, presumably areas that are in proximity to anopheline breeding sites.^7^ Delineating high-risk features in a local context has important applications for malaria control program officers who must decide how to allocate resources or where to conduct active or reactive case detection. On the basis of these findings, targeting households in the 80th percentile of malaria risk would require malaria control directed to fewer than one in four households in the district.^7^ Risk maps were later used to model approaches to enhance the efficiency of reactive case detection through selective screening of secondary households based on their proximity to high-risk environmental features such as streams.^16^

Human movement contributes to the propagation, importation, and spread of malaria parasites.^18^^,^^19^ We mapped population movement over the course of 1 year to understand how human mobility might contribute to sustained, low-level transmission in Choma District.^20^ Sixty-nine adult research participants were equipped with personal GPS loggers for up to 4 weeks. Long-distance travel was more common during the dry season when malaria transmission is lowest, potentially reducing the risk of imported malaria because of this seasonality. There was little movement observed between high- and low-risk areas, with residents of low-risk areas tending to remain in those areas.^20^

### Reactive case detection.

With the decline of malaria transmission in Southern Province, the NMEP targeted the area for elimination. The ICEMR conducted a series of studies of the design, implementation, and impact of reactive case detection strategies designed to interrupt transmission. A pilot project of an RDT-based RTAT program was done in the pre-ICEMR period that confirmed a higher concentration of *P. falciparum*–infected individuals in index case households compared with randomly selected households from the same communities (8 versus 0.7% prevalence by PCR, *P* = 0.006) and a small but nonnegligible proportion of individuals (2%) were gametocytemic as measured by *pfs25* transcripts.^21^ Rapid diagnostic tests missed half to three-quarters of PCR-detectable infections in this setting.^21^

Geospatial, parasite prevalence, and malaria incidence data collected by the ICEMR were then used to simulate RTAT in the study area according to different assumptions about the background transmission intensity and different screening radii from the index household.^11^ The simulations predicted that for low to moderate transmission areas, a screening radius of 500 m was required to identify 75% of cases even with complete follow-up of cases.^11^

In 2013, Zambia implemented RTAT in Choma District and other low transmission areas throughout the country. The program applied a screening radius of 140 m from the index household and the ICEMR assessed the implementation of the program. Within the study area, a wider radius of 250 m was used to study the influence of screening radius size on RTAT efficiency and effectiveness. Within the first year of RTAT in Choma District, community health workers followed-up only one-third of eligible index cases.^12^ Of the index households that were visited, one in three household members, on average, were not present at the time of the visit.^12^ Health worker teams often screened households beyond the intended 140-m radius, introducing additional inefficiencies into the program.^12^ Updated simulations of RTAT and simulations of focal drug administration (i.e., presumptive treatment of index and secondary household members without screening) performed poorly even when assuming complete follow-up of index households within the 140-m radius, reaching only 22% or 57% of the total infected population, respectively.^12^

Overall, RTAT reduced malaria in the index and surrounding households over time, but not to a meaningful extent.^22^ Cases tended to cluster within a household but not within the screening radii and only 11% of index households had at least one additional case.^10^^,^^16^ Of the total cases detected by RTAT, ∼60% were within the case household, ∼10% were within a 140-m radius of the household, and ∼30% were between 140 and 250 m of the household.^10^ Rapid diagnostic tests were insufficiently sensitive to detect the asymptomatic reservoir, missing more than half (55%) of PCR-positive cases.^10^ These low-level infections, invisible to RDT but detectable by PCR, were observed to persist between visits spaced as far as 90 days apart.^22^ A sizeable proportion (27%) of those with sub-RDT parasitemia—mostly older children and young adults—tested positive for gametocytemia by *pfs25* transcripts and therefore were likely to contribute to the infectious reservoir.^23^

Parasite genotyping confirmed that RTAT succeeded in identifying related cases, demonstrating that in principle RTAT is a logical approach to malaria control in transmission foci, notwithstanding the challenges in implementation and insufficient sensitivity of RDTs to detect asymptomatic, low-parasitemia infections.^24^

### Serosurveillance.

Antibody signatures in the human population reflect historical patterns in malaria transmission that can be used to assess the impact of control measures and document progress toward elimination. Gamma immunoglobulin to whole parasite lysate was measured in serum collected during the period of malaria decline (2007–2009) to investigate the ability of serological data to detect relatively short-term changes in local malaria endemicity.^25^ As malaria transmission receded over the 3 years, households with seropositive individuals increasingly clustered in ecologically high-risk areas (classified according to geospatial risk maps, described earlier), demonstrating the ability to predict the geographic trajectory of malaria elimination at small spatial and temporal scales when geospatial and epidemiological data are leveraged.^7^^,^^25^

A more recent ICEMR study used a high-throughput protein array platform to facilitate serological testing across 500 *P. falciparum* and 500 *P. vivax* antigens.^26^ Multiplex serology had the same main limitation of earlier methods—the difficulty of antibody signatures to indicate accurately whether prior infection was recent or remote—but serological indicators in children were found to track with transmission intensity, suggesting that high-throughput serosurveillance of children living in malaria endemic regions could yield useful data for tracking malaria transmission dynamics over time.^26^

### Genetic epidemiology of malaria.

Early ICEMR studies of parasite genomics that relied on microsatellite barcoding and amplicon deep sequencing identified related parasites across transmission seasons and focal transmission networks centered around index case households.^22^^,^^24^ These findings implicated autochthonous malaria transmission in Choma District.

A subsequent analysis of parasite isolates from 2013 to 2018 using multilocus genotyping at > 1,800 sites along the parasite genome revealed a more comprehensive picture of malaria transmission within the district with four key findings.^27^ First, highly related parasites were seen across multiple years—including during the dry season—corroborating the earlier studies and confirming that persistent low-level transmission in Choma District is sustained in part by locally persistent reservoirs that survive through the dry season. Long-term asymptomatic carriers, hidden intact parasites in the spleen, or even dormant *P. falciparum* stages have been proposed as the means by which malaria is sustained during ebbs of mosquito abundance.^28^^–^^30^ Second, there were isolated clusters of parasite strains with no genetic relatives within the overall parasite population, consistent with the importation of genetically distinct parasites. The isolated clusters spanned multiple transmission seasons, suggesting that imported parasites can readily establish themselves in the existing transmission environment. Third, genetic relatedness was higher among samples originating from health facilities in close proximity, implicating focality and geography as relevant features of transmission networks even at a relatively small spatial scale (2,000 km^2^). This mirrors our earlier finding of parasite relatedness across individual RTAT chains.^24^ Fourth, the complexity of infection (number of distinct clones in a single infection) oscillated over time. Most infections were monoclonal, but clonality increased as transmission intensity increases—that is, as health center cases increased—then reverted to mostly monoclonal infections. Low complexity of infection is expected in low-transmission settings; the increase in polyclonality during high-transmission months is consistent with higher EIR and suggests a greater biodiversity of parasites in the human population, providing greater opportunity for genetic recombination.

Additional ICEMR genetic studies were conducted to monitor for *P. falciparum histidine rich protein 2* (*Pfhrp2*) deletions in parasites and glucose-6-phosphate dehydrogenase (G6PD) deficiency in the human population. Parasites were analyzed by PCR amplification for evidence of *Pfhrp2* gene deletion, which renders them undetectable with current HRP2 antigen-based RDTs, but none were found.^3^^,^^31^ Low-transmission Choma District is, in principle, a prime area for the deployment of post-treatment single low dose (SLD) primaquine for gametocyte clearance^32^ but the epidemiology of G6PD deficiency, which predisposes to primaquine-induced hemolytic anemia, is poorly characterized in Zambia with only one prior survey of 200 newborns from the 1980s.^33^ We genotyped 137 adults and identified G6PD deficiency (A-type) in 15% of males while none of the females were carriers.^17^ A small number of trials to date have demonstrated the safety of SLD primaquine in areas of southern Africa and Central America with similar prevalence of G6PD deficiency, suggesting that SLD primaquine is safely deployable in Choma District.^34^^–^^36^

### Vector bionomics.

When the ICEMR began working in conjunction with MRT in Choma District in 2010, the only recognized vector in the region was *Anopheles arabiensis.* This population of *An. arabiensis* was documented as largely anthropophilic with a human blood index of 0.94 to 0.96 based on indoor collections. Human landing catches indicated that the population is active overnight from 18:00 to 06:00 both indoors and outdoors, with peak biting occurring from 23:00 to 03:00.^1^ The work of the ICEMR has expanded the bionomic knowledge of this species, including the detection of a high (19%) frequency of multiple-host feeding on humans, which decreased to 9% after ITN distributions.^37^^,^^38^

Although the feeding preference of *An. arabiensis* has remained largely endophagic, ICEMR studies have shown that the blood feeding rate of this species was previously underestimated by up to 10% and host DNA was detected in up to 11% of visually unfed *An. arabiensis*.^39^ When both visually unfed and fed anophelines were tested using PCR-based methods, the human blood index fell from 0.96 to 0.87 as more nonhuman blood meals were detected.^39^ When collections were extended to both indoor and outdoor traps, only 65% of *An. arabiensis* specimens were captured indoors, and the human blood index decreased even further to 0.70, demonstrating more behavioral plasticity in the species than previously thought.^40^ Although indoor counts of *An. arabiensis* have dramatically declined over the past decade with the introduction of ITNs, seasonal transmission still occurs, indicating that some *An. arabiensis* mosquitoes may avoid indoor interventions and may be contributing to malaria transmission outdoors.

Although *An. arabiensis* was considered the only recognized malaria vector in Choma District before the ICEMR began investigations, significant numbers of *An. squamosus* and *An. coustani* were detected by human landing catches both indoors and outdoors and high proportions were found to have fed on humans (86% for *An. coustani* and 65% for *An. squamosus*), although none were positive for sporozoites.^41^ In 2015, the ICEMR detected the first *An. squamosus* positive for *P. falciparum* sporozoites in this region, followed by sporozoites detected in *An. rufipes* and *An. coustani* in 2016 and 2017, respectively.^40^^,^^42^ Proper identification of these species relies on a combination of morphology and molecular assays, which can be challenging when limited genetic references are available.^43^ Studies by the ICEMR have provided extensive genetic data for these anophelines to public databases. These studies have reported evidence that *An. squamosus* and *An. coustani* populations in Zambia each consist of at least two genetic clades, alluding to yet undescribed species complexes.^44^^,^^45^ In these studies, *An squamosus*, *An. coustani*, and *An. rufipies* were largely found to be zoophilic and exophilic, making them difficult to target with current entomological interventions.^40^^,^^45^ The identification of understudied vectors and their distinct bionomics relative to the predominant vector, *An. arabiensis*, has potentially major implications for vector control and malaria elimination in southern Zambia. Additional studies to further characterize their genetics, behaviors, and susceptibility to insecticides are ongoing.

### Discussion.

Ten years of observation and analysis by the ICEMR in Choma District, an area of sustained low-level malaria transmission targeted for elimination, yielded key findings with context-specific and generalizable applications. In broad terms, malaria appears to be sustained in Choma District by both the trans-seasonal persistence of local parasites and the importation and subsequent establishment of parasites from outside the study area, although the relative contribution of each is not known and likely varies across transmission seasons.

Insecticide treated nets were shown to be effective but, despite high coverage (> 80% household ownership), usage was 55% lower in children than in adults. School-age children, who reported the lowest ITN use, had the highest carriage of gametocytes, representing an important transmission reservoir. Similarly, individuals who tested positive for asexual parasites by PCR but negative by RDT were found to have a nonnegligible prevalence (2%) of gametocytemia. These are individuals who would go undetected by active or reactive case detection.

Malaria risk maps generated from time series and geospatial data performed well in predicting clustering of case households during the short-term decline in malaria transmission in 2007–2009 and could be prospectively used for tailoring active or reactive case detection strategies.

The ICEMR showed that RTAT succeeds in identifying chains of malaria transmission but effective implementation of RTAT is extraordinarily difficult. Models predicted that even with a screening radius of 500 m from an index case household, only 75% of secondary cases would be detected assuming complete follow-up and a perfectly sensitive screening test. The RTAT program implemented in Choma District imposed a screening radius less than half that size, 140 m, and teams followed up on only one-third of eligible index cases and missed on average one in three household members at the beginning of the program. Predictably, RTAT did not succeed in reducing malaria transmission to a measurable extent within the study area.

The sensitivity of commonly available *P. falciparum* RDTs was extremely poor in detecting asymptomatic, low-density infections. Reactive test-and-treat and other active case detection strategies that rely on RDTs for screening will therefore fail to interrupt transmission by leaving a large untreated reservoir of infected individuals. The sensitivity of RDTs for reactive case detection was 45% and for community-based active case detection it was as low as 17% during the low-transmission season when residual parasites from the prior transmission season can persist to cause a resurgence of malaria in the next season, as shown in ICEMR studies of parasite genomics.

The discovery that the understudied vectors *An. squamosus*, *An. coustani*, and *An. rufipes* harbor sporozoites and may contribute to *P. falciparum* transmission has important implications for vector surveillance and control in Choma District and elsewhere in Zambia where these species have since been reported. Unlike the main vector *An. arabiensis*, these three species are zoophilic and exophilic, allowing them to avoid indoor-based interventions such as ITNs and IRS. They have now been reported elsewhere in Zambia.

Other important contributions of the ICEMR in Choma District include demonstration of the utility of serosurveillance for documenting short-term changes in malaria transmission, the absence of prevalent *Pfhrp2* deletions in the parasite population supporting the continued use of current RDTs, and a moderate prevalence of G6PD deficiency at a level that does not seem to preclude the deployment of SLD primaquine. Ongoing studies include the genetic, epidemiologic, and temporospatial characterization of transmission chains on a fine spatial and temporal scale, ecological predictors of long-term malaria periodicity, molecular entomology, and the use of a real-time data visualization mobile phone application used by local healthcare workers to help target malaria control interventions throughout the district.

## REFRACTORY HIGH-LEVEL MALARIA TRANSMISSION IN NORTHERN ZAMBIA

Nchelenge District, an area of holoendemic malaria, is located in Zambia’s northern province of Luapula situated along Lake Mweru bordering the Democratic Republic of the Congo (DRC). The local geography consists of miombo woodland and wetlands fed by the Luapula River and its tributaries. Residents reside mainly in sundried brick and thatch houses and subsist on farming and fishing. The main malaria vector is *An. funestus* that peaks during the dry season. *An. gambiae* is also present but in lower numbers, mainly in the lakeside area.^46^^,^^47^ *P. malariae*, *P. ovale*, and *P. vivax* rarely cocirculate with *P. falciparum* (< 1–6% mixed or monoinfections).^48^

### Methods.

ICEMR research activities in Nchelenge District started in 2012 in partnership with the Tropical Diseases Research Centre in the wake of a 6-year scaleup of malaria control in the region. Starting in 2006, the NMEP introduced RDT- and ACT-based case management to the district, distributed more than 300,000 ITNs, and from 2008 onward conducted annual IRS campaigns first targeted to the densely populated lakeside area and later expanded inland.

ICEMR passive and active surveillance are done similarly to that in Choma District. The 15 district health centers contribute case data weekly. Monthly aggregated data on hospital admissions for malaria and malaria-attributable deaths grouped by age (< 1 year old, 1 to < 5 years old, ≥ 5 years old) are collected from the district hospital for surveillance of severe malaria. A hospital-based retrospective cohort of children hospitalized with malaria (*n* = 1,115) contains data on demographic characteristics, clinical features, laboratory values, treatments, comorbidities, length of stay, and survival.

Active surveillance, ongoing since 2012, consists of cross-sectional (*n* = 8,543 participants) and longitudinal (*n* = 560 participants) household surveys. Households enrolled in the longitudinal cohort were visited bimonthly from 2012 to 2017 and repeated cross-sectional surveys have been done continually since 2012. Data were collected as described above for Choma District. PCR testing for malaria parasites was done during the first round of the ICEMR in 2012–2017. A human movement study using GPS loggers recruited 82 participants from within longitudinal households. Entomological surveys and collections by CDC light traps were done in all households (*n* = 2,034) each visit overnight for 1 night.

Two prospective trials were done in Nchelenge District leveraging the ICEMR: a therapeutic efficacy study of artemether-lumefantrine carried out as an ICEMR special project (PACTR201905783261453)^49^ and a Johns Hopkins Malaria Research Institute–sponsored randomized controlled trial of artemether-lumefantrine versus dihydroartemisinin-piperaquine for uncomplicated falciparum malaria in children (NCT04009343).

### Epidemiological trends.

**Figure 2.**
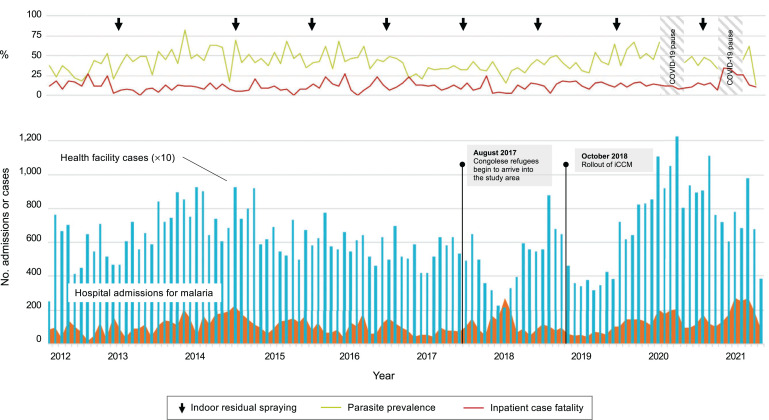
Malaria indicators in Nchelenge District showing persistently high burden despite control efforts. (**Top**) Parasite prevalence by rapid diagnostic test, inpatient case fatality, and timing of indoor residual spraying (IRS) campaigns, which coincides with the start of the rainy season. In 2013, an insecticide treated net distribution was done in place of IRS. (**Bottom**) Monthly hospital admissions for malaria and health facility cases across all age groups. Health facility cases are shown at 1/10th scale. Data begin April 2012. This figure appears in color at www.ajtmh.org.

With the implementation of the first National Malaria Control Strategic Plan in 2006, malaria in Zambia substantially declined throughout most of the country but Nchelenge District continued to experience a high incidence of malaria despite a scale-up of measures that included the deployment of RDTs and ACTs for case management, distributions of ITNs, annual targeted IRS, and, more recently, the training of more than 300 new community health workers as part of an expansion of Zambia’s integrated community case management (iCCM) program (Figure 2).^50^

Health facility cases for malaria were 158 to 340 cases per 1,000 population per year from 2012 to 2020, with 50% of cases occurring in children younger than 5 years. There were no differences across years either in case incidence or the age distribution of cases. Between 1,000 and 2,000 patients are admitted for severe malaria each year to the district hospital. Malaria accounts for approximately one-third of pediatric hospital admissions and 40% of pediatric inpatient deaths.^51^ Hospitalizations for malaria ranged from 3.3 to 7.7 per 1,000 population per year in the same period, with 60% of cases in children younger than 5 years. Inpatient case fatality for malaria was 12% in children under 5 years and 11% in children older than 5 years and adults. The median age of children hospitalized with malaria has increased slightly but significantly over 2017 to 2020 from 23 to 29 months (*P* < 0.01).

Community parasite prevalence by RDT ranged from 30% to 50% each year from 2012 to 2020 and by PCR from 18% to 45% from 2012 to 2017. In adults, parasite prevalence measured by RDT (21%) was similar to PCR (24%) but in both children under 5 years and school-age children, RDT overestimated parasite prevalence by a factor of 1.4 times, presumably due to residual parasite antigenemia from recently cleared infections and reflecting the high incidence of malaria in these age groups.

Malaria indicators were affected by the influx, beginning in 2017, of more than 15,000 refugees from a high malaria transmission region of the DRC who were resettled in Nchelenge District. A refugee settlement health facility was established and started to contribute data to the ICEMR passive surveillance system in October 2018. Annual case incidence in refugees was an estimated 654 to 764 cases per 1,000 population per year in 2019–2020 (not reflected in the preceding data) and refugee children experienced higher inpatient mortality than nonrefugee children (25 versus 13% for 2017–2020, *P* < 0.001).^52^

Overall, health facility case incidence, community parasite prevalence, hospitalizations for malaria, and inpatient malaria-attributable case fatality in Nchelenge District remain high and essentially unchanged despite control efforts.

### Risk factors.

Different environmental and entomological features in Nchelenge District translate to differences in risk factors for malaria compared with Choma District in southern Zambia, particularly regarding the seasonality of malaria transmission. During the rainy season in Nchelenge District, proximity to roads was associated with malaria risk.^53^ Temporary vector mosquito breeding sites in stagnant water that accumulates in tire tracks and divots along the roadside are one possible explanation. In contrast, during the dry season living in proximity to streams was associated with higher malaria risk.^54^ Interestingly, greater stream flow rates lowered the risk, suggesting that mosquito breeding sites along stream beds may be washed out by currents.^54^ Other risk factors were less context-specific: younger age, male sex, low-quality housing, living at lower elevation, higher rainfall, warmer weather, and lower educational attainment of the head of household were associated with higher malaria risk.^53^^–^^55^ Using a mosquito net and living in a household with more nets per occupant were protective against malaria.^53^^–^^55^ In hospitalized children with malaria, blood stockouts and referral from a village or health center further from the hospital were associated with higher inpatient mortality.^56^ Whole blood transfusion was associated with improved survival in children with severe malarial anemia, and transfusion using whole blood stored > 28 days was no better than no transfusion.^57^

Human movement studies showed frequent monthly travel between relatively high and low risk areas, beyond expected mobility for farming and fishing, and between sprayed and unsprayed zones. Half of participants traveled at least once between the lakeside and inland areas and nearly three-quarters traveled between sprayed and unsprayed areas.^58^

### Targeted indoor residual spraying.

Targeted IRS has been conducted yearly in Nchelenge District since 2008 with little effect on the overall incidence or prevalence of malaria. Spraying is done at the onset of the rainy season, starting in November and ending in December, initially targeted to the densely populated lakeside area but with expanded coverage over the past several years. Inland areas, where ICEMR data show malaria risk to be higher than the lakeside independent of spraying,^54^ were excluded before 2017 due to difficult-to-traverse roads and insufficient funds to spray all households in the district. Spraying was supervised by the U.S. President’s Malaria Initiative VectorLink Project, and the proportion of targeted households that participated was > 80%.

During the 2011 and 2012 spray seasons, Nchelenge District relied on a combination of ITNs and IRS with carbamates, transitioning to ITNs alone for the 2013 spray season. From 2014 to 2016, IRS with the organophosphate insecticide pirimiphos-methyl (Actellic™) reduced parasite prevalence in the targeted areas by 28% during the rainy season after IRS but had little impact on dry season prevalence or in the overall study area.^54^ In the targeted areas, there was a moderate decrease in unsprayed households implicating both direct and indirect effects of IRS. IRS did not reduce the number of *An. funestus* in a household, but there was a moderate but statistically insignificant reduction in *An. gambiae* counts. There was no indirect benefit of IRS on any household level outcome and no difference in health center cases in sprayed versus unsprayed areas, although there was a decrease in the overall number of malaria cases during the study period.

During the 2019 and 2020 IRS campaigns, spraying was conducted using Fludora® Fusion, a long-acting insecticide formulation containing deltamethrin and clothianidin. However, no change in rainy season parasite prevalence comparing sprayed to unsprayed households was estimated compared with previous years during which Actellic® 300CS was used (unpublished data), and living in a house sprayed with either insecticide was not associated with decreased parasite prevalence during the rainy or dry season. In contrast, each 10% increase in the percent of households sprayed was associated with a 4% to 5% decrease in parasite prevalence, suggesting a predominantly community-level protective effect of IRS.

### Molecular epidemiology.

The ICEMR’s molecular epidemiological studies of malaria in Nchelenge District have characterized the transmission dynamics, reservoirs of infection, population exposure to *P. falciparum*, parasite drug resistance, and mosquito insecticide resistance. Consistent with other areas of intense transmission, parasite genetic diversity in Nchelenge District is high and infections tend to feature more than one parasite clone.^26^

Differences in genetic diversity and polyclonality over geography and time reflect the underlying epidemiology of malaria and can provide a sensitive means for assessing the impact of control measures and characterizing geographically distinct parasite populations. Analyses of parasite haplotypes from Nchelenge District and from Haut-Katanga Province, DRC, across the border showed genetically indistinct populations consistent with a contiguous transmission zone and implicating potential importation of parasites into Nchelenge District from DRC where there is less malaria control activity.^26^ An analysis of parasite haplotypes before and after an ITN distribution in 2016 and 2017 in Haut-Katanga failed to show any change in genetic diversity, arguing against a major impact on transmission.^26^ Consistent with the high intensity of malaria transmission across the region, and in contrast to seroprevalence studies in Choma District, antibody signatures of malaria in Nchelenge District study participants were elevated and equivalent across all age groups (including young children) in samples collected in 2015, corroborating the lack of a significant impact of control measures up to that time.^59^

Similar to Choma District, molecular studies in Nchelenge District found that school-age children have both the highest prevalence of *P. falciparum* infection^54^ and highest gametocyte carriage (unpublished data), and they were least likely to use an ITN,^60^ representing an important reservoir of infection and important target for control efforts. Parasite genetic studies also identified a high prevalence of antifolate drug resistance similar to neighboring areas of southern and central Africa,^49^ whereas parasite clearance studies in 2014–2015^49^ and 2019–2020 (unpublished data) showed no evidence of delayed parasite clearance to implicate ACT resistance. Molecular analysis of samples up to 2 weeks after treatment showed evidence of residual parasitemia and gametocytemia by transcripts of *pfs18*, *pfsbp1*, and *pfs25*.^61^ In addition to silent reservoirs of *P. falciparum* in older children and adults, post-treatment residual gametocytemia adds an additional complication to malaria control and emphasizes the need for continued drug and vaccine development that considers their impact on gametocytes.

### Vector bionomics.

Relatively little was known about the main malaria vectors in Nchelenge District before the ICEMR. The ICEMR has performed extensive cross-sectional and longitudinal entomological studies across the district to reveal that the entomological patterns and year-round malaria transmission in Nchelenge District are largely driven by its particular ecology and vector bionomics. The western edge of the district is bordered by Lake Mweru, which separates Nchelenge District from the DRC. *An. gambiae* is present year-round and is found predominantly along the lake where ephemeral pools are likely to serve as the primary larval habitat.^46^^,^^47^^,^^62^ In contrast, extensive swampy habitats innervated by streams located inland from Lake Mweru provide year-round larval habitats for *An. funestus*, the major malaria vector in Nchelenge District. This is exacerbated during the dry season when the water levels are lower and move slower, producing large numbers of *An. fune*stus.^46^^,^^47^^,^^62^ *An. fun*estus counts consistently vastly outnumber the *An. gambiae* counts, with 2012 dry season EIRs calculated as 41.5 and <1 infectious bites per person per 6 months for *An. funestus* and *An. gambiae*, respectively.^46^ Even during the rainy seasons in 2012–2013, *An. funestus* appeared to contribute more to transmission with respective EIRs at 3.7 to 39.6 and <1 to 5.9 infectious bites per person per 6 months in *An*. *funestus* and *An. gambiae*.^46^ The ICEMR has also investigated host preferences and multiplicity of infection of these species and found a human biting index of 1.0 for both *An. funestus* and *An. gambiae*, and a mean multiplicity of infection of 6.4 *P. falciparum* genotypes among infected vectors, high compared with other studies across Africa and consistent with high malaria burden.^63^

Vector control in Nchelenge District has consisted of ITN distributions in 2014, 2017, and 2020 and IRS campaigns since 2008 conducted at the beginning of the rainy season each year except 2013. The ICEMR performed impact evaluations of 3 years IRS using pirimiphos-methyl and found a modest decrease in *An. funestus* populations but no impact on *An. gambiae* populations.^62^ Studies evaluating the effect of 5 years of pirimiphos-methyl and one year of Fludora^®^ Fusion are ongoing.

Higher rates of using a mosquito net also appear to decrease counts of both vectors in households.^64^ However, after three mass ITN distributions and annual IRS, malaria transmission remains holoendemic. The ICEMR is currently investigating multiple hypotheses regarding the lack of impact, notably that the pre-rainy season timing of IRS does not reduce the high abundance of dry season anophelines and that *An. funestus* populations may be foraging outdoors. The necessity for continued surveillance, intervention evaluation, and the addition of new vector control measures is starkly evident in this holoendemic setting.

Annual insecticide resistance studies have been conducted since 2012. Resistance to pyrethroids and carbamates is widespread in *An. funestus* which remains highly susceptible to dichlorodiphenyltrichloroethane (DDT) and organophosphates. The *An. gambiae* population is highly resistant to pyrethroids and DDT, moderately resistant to the carbamates, and remain highly susceptible to organophosphates.^65^

### Discussion.

Malaria in Nchelenge District remains highly prevalent and is the most common cause of hospital admission and death in children despite a decade and a half of malaria control efforts that consisted of RDT- and ACT-based case management, targeted IRS, and mass ITN distributions. The abundant mosquito population of *An. funestus* during the dry season, after IRS efficacy has worn off, is the main driver of transmission. The vector is known to feed outdoors, compounding challenges to vector control efforts that currently rely on indoor interventions, the limitations of which are also highlighted by human movement studies that found Nchelenge District residents spend a significant amount of time outside during peak biting times.^58^

Targeted IRS as a malaria control tactic in Nchelenge District proved effective in principle over a 3-year period after the introduction of pirimiphos-methyl—with reductions in parasite prevalence seen during the rainy season in individually sprayed households and nearby unsprayed households—but not in practice over the succeeding years. The timing of IRS at the onset of the rainy season does not target *An. funestus* at its peak and the initial exclusion of inland areas, where malaria risk is highest and larval sites are thought to proliferate during the dry season, left a critical gap. The misaligned timing of IRS relative to *An. funestus* vector abundance may explain why IRS in Nchelenge District has had little measurable impact. Pyrethroid resistance may further compound the problem by limiting ITN efficacy.

Other remaining challenges to malaria control in Nchelenge District are supply chain interruptions and its proximity to the DRC, where malaria control operations are less intensive and parasites were seen through genetic analyses to circulate cross-border. Cross-border cooperation and co-investment in malaria control can mitigate the latter, and excess death due to stockouts of essential medical supplies can be curtailed through improved logistics.

## BORDER MALARIA IN MESOENDEMIC ZIMBABWE

Mutasa District is located in Manicaland Province on Zimbabwe’s eastern border with Mozambique. It is situated partly on the Highveld, the southern Africa inland plateau (elevation ∼1500 m), which receives high rainfall. Malaria caused by *P. falciparum* peaks during the rainy season from November to April and is transmitted by *An. funestus*, with most cases concentrated in the lower elevation Honde Valley area along the border.^66^ The ICEMR has conducted active and passive surveillance in Mutasa District since 2012 in partnership with the Biomedical Research and Training Institute, Africa University, and National Institutes of Health Research. Methodologies are similar to those for the two Zambian sites.

### Epidemiological trends.

**Figure 3.**
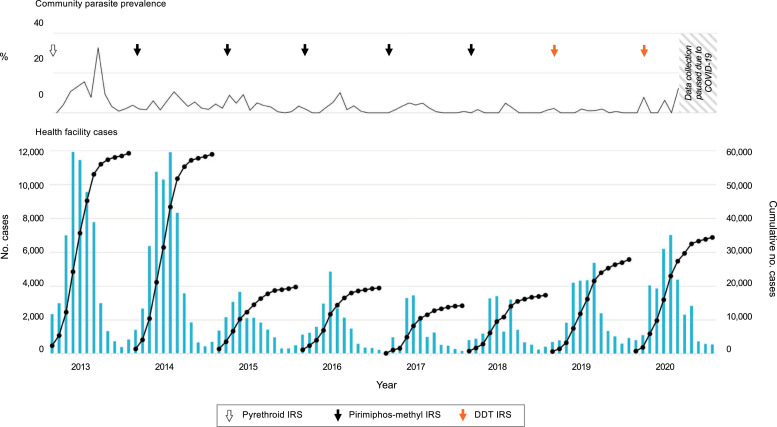
Malaria indicators in Mutasa District showing persistent seasonal malaria with sustained reductions after indoor residual spraying (IRS) with pirimiphos-methyl starting in 2014 until 2018 when the insecticide was changed to dichlorodiphenyltrichloroethane (DDT) with a subsequent rise in parasite prevalence and health center cases. (**Top**) Parasite prevalence by rapid diagnostic test and timing of IRS. (**Bottom**) Health facility malaria cases per month (bars) and cumulative cases (curves) from October to September the following year. This figure appears in color at www.ajtmh.org.

Zimbabwe aims to eliminate malaria by 2030 but continues to experience a mesoendemic level of transmission in Mutasa District and similar areas of lower elevation (∼900 m) bordering Mozambique, where malaria occurs across all age groups with the greatest burden in older children and adults.^66^ Cases peak during the rainy season from November through April with an average parasite prevalence of 4.8% compared with 1.4% during the dry season (Figure 3).^67^ Higher rainfall and night temperature are associated with increased health facility incidence,^68^ and lower elevation is associated with higher malaria risk at the household level in the rainy season and higher health facility incidence year-round.^67^^,^^69^

Transmission is thought to be increased by individuals who travel routinely to and from adjacent Manica Province, Mozambique. Consistent with this, geostatistical analyses of active ICEMR surveillance data showed greatest risk along the Mozambique border after controlling for environmental and ecological features, and for every 1-km increase in proximity to the border the odds of parasitemia increased 16% for individuals and 14% at the household level.^67^^,^^69^

### Assessments of malaria control.

Vector control in Mutasa District has relied on IRS for the past 6 decades and ITNs over the past 2 decades.^66^^,^^70^ Both IRS and ITN were associated with reductions in local malaria burden but have been vulnerable to the rise of insecticide resistance and insufficient ITN coverage and adherence. As of 2014, an estimated 92% of the district’s population was covered by IRS,^71^ while access to and use of ITNs has been limited in comparison. From 2012 to 2017, 70% of ICEMR study households owned at least one ITN, but only 40% had one available ITN for every two people, and only 32% of participants reported sleeping under an ITN.^60^ Use of an ITN was associated with a 50% decrease in the odds of parasitemia despite local mosquito resistance to pyrethroids.^67^ Promisingly, full susceptibility to deltamethrin in the presence of piperonyl butoxide (PBO) was demonstrated in 2014, suggesting an opportunity for improved malaria prevention with PBO-treated ITNs should pyrethroid nets lose their efficacy.^65^

Insecticide resistance similarly threatens the efficacy of IRS. In 2013 and 2014, local *An. funestus* were found by the ICEMR to be resistant to pyrethroids and carbamates, prompting the transition from pyrethroids to organophosphates for IRS.^65^^,^^68^ After the first application of pirimiphos-methyl in 2014, health facility case incidence of malaria declined 38% in sprayed areas after the first round of IRS, but further reductions were not observed in subsequent years of spraying.^68^

Clinical malaria case counts remained down from 2014 until 2018 when the district switched to DDT and the U.S. President’s Malaria Initiative transitioned operations to the Zimbabwe National Malaria Control Program. Since that time, annual clinical cases and rainy-season parasite prevalence in 2019–2020 increased over 2017–2018 from an average of 15,805 cases per year to 31,148 cases per year and from 1.1% to 4.4% parasite prevalence.

Case management has undergone multiple changes over the past 2 decades. Until 2003, chloroquine (CQ) was used in Zimbabwe to treat uncomplicated cases. In response to parasite drug resistance to CQ, Zimbabwe replaced monotherapy with the combination CQ and sulfadoxine-pyrimethamine in 2003 and, subsequently, artemether-lumefantrine in 2008. The ICEMR has monitored CQ resistance in Mutasa District following the transition away from CQ monotherapy. In 2003, resistance was widespread with *CQ resistance transporter* gene (*pfcrt*) mutants detected in 67% of sampled parasites.^70^ By 2013, *pfcrt* mutants were present in only 3% of samples, and by 2017–2018 all *P. falciparum* tested were *pfcrt* wild-type, providing documentation of the reversion to CQ-susceptible *P. falciparum* in Mutasa District.^72^

### Discussion.

Zimbabwe aims for malaria elimination within the decade but continues to see low to moderate transmission along its lowland border areas climbing in recent years. The usual package of IRS with pirimiphos-methyl and ITNs reduced transmission to a measurable extent when first introduced into the study area, attesting to their efficacy in this context, but the change from pirimiphos-methyl to DDT and the handover of IRS program management from PMI to NMEP corresponded to an increase in transmission in the years that followed: health facility cases doubled and parasite prevalence quadrupled. The causes of the increase are currently under study by the ICEMR and may include reduced IRS efficacy and/or coverage (due to insecticide resistance, quality of the chemicals, changes in program management, or other factors), climate-related changes in transmission, or cross-border importation among others. For Zimbabwe to resume progress toward elimination, malaria control interventions will need to be increasingly tailored to border areas. In addition to ensuring high coverage with IRS and ITNs, strategies such as coordinated cross-border malaria control programming, guaranteed healthcare access to nationals and non-nationals in border areas, and traveler screening at border crossings could be integrated into current efforts.

## CONCLUSION

The ICEMR progam was established by the National Institute of Allergy and Infectious Diseases to conduct interdisciplinary research and expand local capacity for the advancement of malaria control. The Southern and Central Africa ICEMR has operated for a decade in Zambia and Zimbabwe across areas of hypo-, meso-, and holoendemic malaria where the selection of control interventions varied according to the local epidemiology, ecology, entomology, resource availability, and policy priorities. Control programs met with scant success despite substantial investments. The addition of a reactive case detection program to existing measures was insufficient to eliminate malaria in hypoendemic Choma District for several reasons, among them the limited sensitivity of RDTs and strained capacity for timely, complete screening. In holoendemic Nchelenge District, targeted IRS had little measurable impact and hospital shortages led to excess deaths from malaria. Mutasa District, in a mesoendemic border area of Zimbabwe, experienced an initial decline in malaria early in the decade after the transition from pyrethroid to pirimiphos-methyl IRS, but in the past 2 years has seen a resurgence. Across multiple sites, asymptomatic reservoirs, especially in older children, persisted through the dry season, complicating efforts to identify and treat cases to interrupt transmission, while exophagy in primary and secondary anopheline vectors confounds current vector control measures that are relegated indoors.

The experience of the Southern and Central Africa ICEMR recalls the history of malaria control programs throughout the past century across the globe, which struggled to enact complex interventions.^73^ More than half a century ago, the final assessment of the Global Malaria Eradication Program and, later, the seminal Garki Project invoked many of the same limitations and challenges, arising in part from failures to sufficiently understand and tailor interventions to local conditions.^74^^,^^75^ Today, the mission of the ICEMR is as relevant as ever given the need for deeper knowledge of the challenges and opportunities particular to different malaria transmission settings at a time when the coronavirus pandemic poses the greatest threat to malaria control in decades and the first-ever malaria vaccine offers perhaps the greatest opportunity in a generation.
